# A Tube Furnace Design for the Oxygen Annealing of a REBCO Superconducting Joint

**DOI:** 10.3390/ma18133053

**Published:** 2025-06-27

**Authors:** Zili Zhang, Chuangan Liu, Yang Gao, Hongli Suo, Lei Wang, Shunzhong Chen, Jianhua Liu, Qiuliang Wang

**Affiliations:** 1 Institute of Electrical Engineering, Chinese Academy of Sciences, Beijing 100190, China; zilizhang0816@vip.126.com (Z.Z.); wanglei@mail.iee.ac.cn (L.W.); liujianhua@mail.iee.ac.cn (J.L.); 2 University of the Chinese Academy of Sciences, Beijing 100190, China; 3 Key Laboratory of Advanced Functional Materials, Ministry of Education, College of Materials Science and Engineering, Beijing University of Technology, Beijing 100022, China; liuchuangan@email.bjut.edu.cn (C.L.); gaoyang@email.bjut.edu.cn (Y.G.); honglisuo@bjut.edu.cn (H.S.)

**Keywords:** REBCO superconducting joint, oxygen annealing, tube furnace, temperature distribution

## Abstract

In this study, we investigated how to design a tube furnace for the oxygen annealing of a REBa_2_Cu_3_O_7−x_ (REBCO, where RE = rare earth) superconducting joint. We confirmed the annealing temperature threshold of REBCO tape *I*_c_ degradation, which was 175C. A heat exchange model that included REBCO tape and a tube furnace was established by using this temperature as the boundary condition. At the same time, the temperature distribution of the REBCO tape in a commercial tube furnace was measured for the calibration of the heat exchange model. The feasibility and accuracy of the model were confirmed by comparing the real measurements and the simulation results. We then optimized the furnace design based on the model according to two criteria: a 20 mm length of REBCO tape should be kept at high temperatures for the oxygen annealing of REBCO joints and the length of tape at temperatures over the *I*_c_ degradation temperature should be as short as possible. The results of this furnace design investigation could help fabricate shorter REBCO superconducting joints, making the magnet more compact and decreasing the length of the Cu stabilizer layer to be removed.

## 1. Introduction

High-temperature superconductors (HTSs), especially of the REBCO type, have been widely used in multiple application scenarios [1,2,3,4,5,6]. Nuclear magnetic resonance (NMR) spectroscopy is one of the most interesting applications. The resolution and sensitivity of NMR are proportional to the magnetic field strength, which promotes the development of extremely high-field NMR, namely over 1 GHz, where it is necessary to use an HTS.

One of the most critical parameters of NMR is magnetic stability, which is typically achieved in persistent mode by using a superconducting joint between the coils. Superconducting joints in low-temperature superconductors (LTSs), such as NbTi and Nb3Sn, are easy to obtain. However, the low critical current of LTSs prevents application at 900 MHz and above [7]. To date, there have been some reports on NMR projects over 1 GHz, such as the 1.02 GHz NMR project in Japan [8], the 1.3 GHz MIRAI NMR plan in Japan [9], a 1.3 GHz NMR project at MIT [10], and the world’s first 1.2 GHz commercial NMR device by Bruker [11]. Among these projects, the 1.3 GHz MIRAI NMR plan is the only one confirmed to use a superconducting joint between HTS coils [9]. Although the persistent mode was used in the 1.2 GHz commercial NMR device by Bruker, it is not clear whether the superconducting joint of the REBCO coil was used [12]. Tian et al. showed that a meter-length REBCO ultra-low-resistance joint might satisfy the requirements of giga-hertz NMR by using the LTS-HTS, but it has not been proven that Bruker uses a similar technology [6].

The first REBCO superconducting joint was obtained in 2014 [13], with a joint resistance as low as 10^−17^ Ω. A crystalline joint obtained with a melted bulk (CJMB) method and intermediated grown superconductors (iGSs) can also reach a resistance lower than 10^−12^ Ω [14,15,16,17]. All of these methods similarly adopt two annealing steps: one is performed at a high temperature (800~900 °C) to join the REBCO tape, and the other is conducted at a low temperature (400~500 °C) under an oxygen atmosphere to perform reoxygenation after high-temperature annealing. Reoxygenation annealing normally continues for tens or even hundreds of hours. The outermost layer of a REBCO-coated conductor is the Cu stabilizer layer, which cannot endure long annealing under oxygen; therefore, the Cu layer should be removed for joint fabrication.

To date, most research on persistent-current REBCO joints has focused on short samples, and only the MIRAI plan shows an actual application in a magnet. In the fabrication of REBCO superconducting joints, the joints must be made at a distance of some tens of centimeters or even meters from the coil to avoid possible damage to the latter due to the long annealing time [17,18]. Reference 17 shows that the superconducting joint was positioned at least 1 m above the magnet, which made the magnet insufficiently compact. The author also mentioned that this was because the furnace used for the joining process required a long terminal length [17]. This furnace problem exists not only in the iGS method, but also in all other superconducting joint methods. Research on a unique furnace design for superconducting joints could allow for greater compactness of the whole magnet design and for the determination of how long the Cu layer, which plays an essential role in stabilization and protection, should be left before removal during joint fabrication.

In this study, a tube furnace design for the oxygen annealing of a REBCO superconducting joint was systematically investigated with both experiments and simulations.

## 2. Experiment

**Figure 1:**
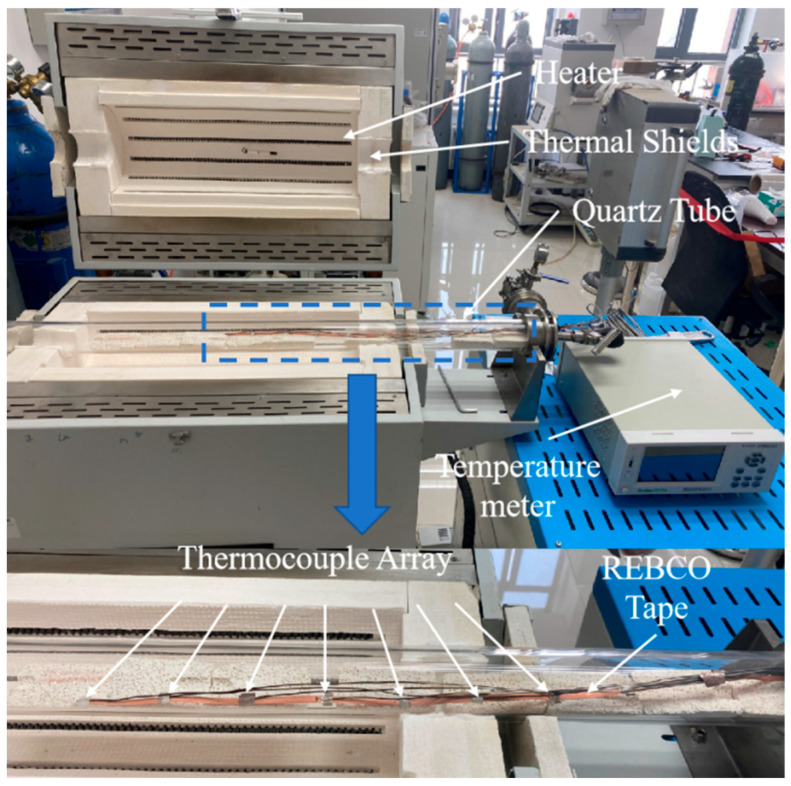
Photo of tube furnace and sample measurement system.

The primary work performed in this study consisted of a tape temperature measurement in a tube furnace and the simulation of the latter based on the measurement results. Figure 1 shows the tube furnace and the sample measurement system. The REBCO tape was positioned in a commercial tube furnace, which allowed for flowing gas. The quartz tube was longer than the furnace part. An eight-channel thermocouple array was used to measure the temperature distribution of the REBCO tape, to which it was tightly attached by using Ag foil; therefore, the thermal conduction path between the thermocouple and the REBCO tape could be ignored. To obtain more temperature data for the REBCO tape, the latter was divided into two parts and separately measured with another eight-channel thermocouple array. The specific heat of the REBCO tape was measured with the sapphire method by using a differential scanning calorimeter (DSC). The tube furnace model was established by using the COMSOL Multiphysics software 6.4 Sweden.

## 3. Results

**Figure 2:**
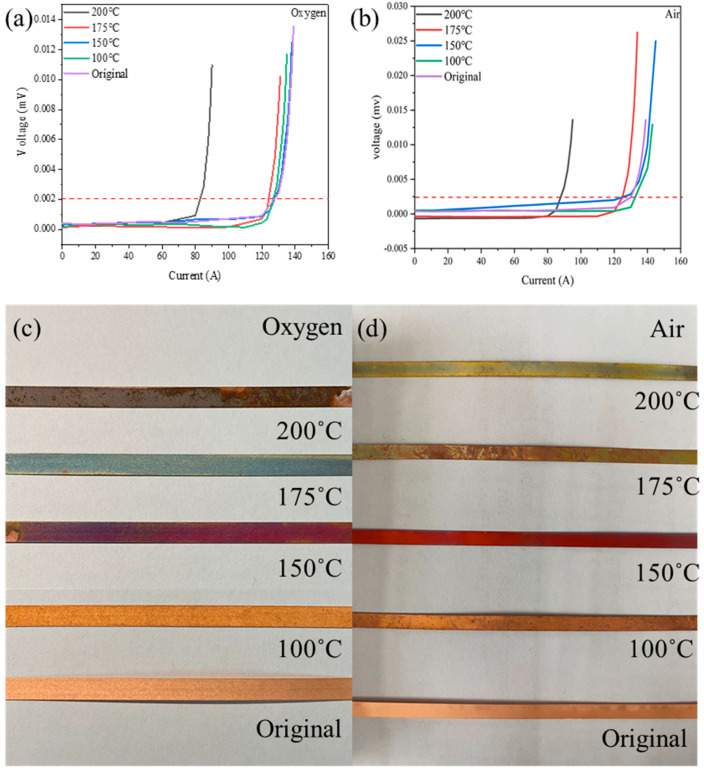
The I–V curves and photos of the REBCO tape sintered at different temperatures for 5 h under oxygen (**a**,**c**) and air (**b**,**d**). The dotted line is the criterion of the *I*_c_ value, which is 1 μV/cm.

The first step of the furnace design was to confirm the temperature threshold that can damage the REBCO tape. Figure 2 shows the I–V curve and a photo of the REBCO tape sintered at different temperatures under oxygen and air. Although the surface color of the REBCO tape started to change at 125 °C under oxygen, *I*_c_ only showed apparent degradation at 175 °C, at which point the surface color was greatly altered. The *I*_c_ values are shown in Table 1. This means that we cannot determine the *I*_c_ degradation only from the color change in the Cu layer. The temperature threshold for air annealing was 150~175 °C, similar to oxygen annealing. In this study, we used 175 °C as the criterion to design the tube furnace. Similar results were also reported in [19,20]. As shown in the results from Lucas et al. and M. Bonura et al., the *I*_c_ value also showed degradation at a temperature of around 200 °C, which is consistent with our work.

**Figure 3:**
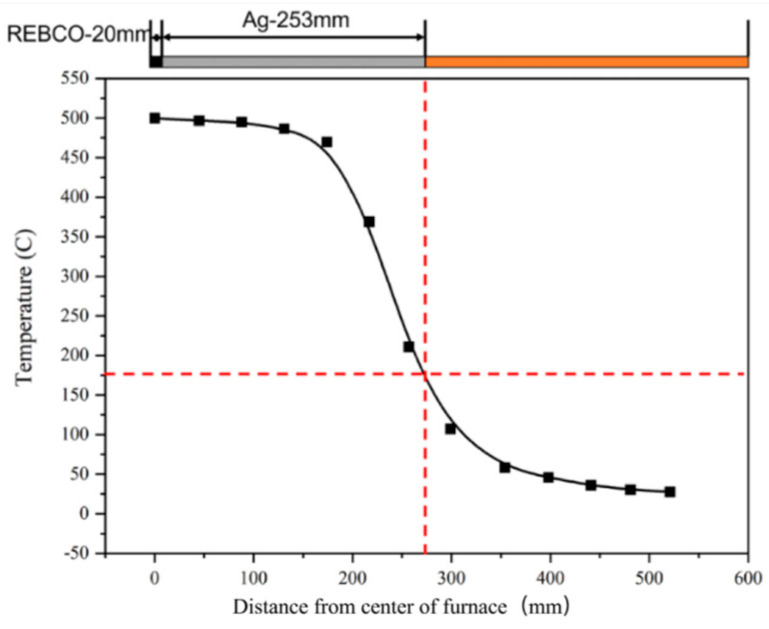
The measured temperature profile of the REBCO tape in the tube furnace set to 500 °C under an oxygen flow of 60 mL/min.

Figure 3 shows the measured temperature of the REBCO tape in the tube furnace set to 500 °C under an oxygen flow of 60 mL/min. The temperature started to decrease sharply at a distance of 174 mm from the middle of the furnace, which fits the furnace temperature distribution shown below. The temperature of 175 °C, set as the threshold of the REBCO tape with the Cu layer, corresponded to 273 mm from the middle. This means that, if the oxygen annealing of a superconducting joint was performed with this tube furnace, except for 20 mm for the joint area, another 253 mm long Cu layer should be removed to maintain the current capability of the REBCO tape.

**Figure 4:**
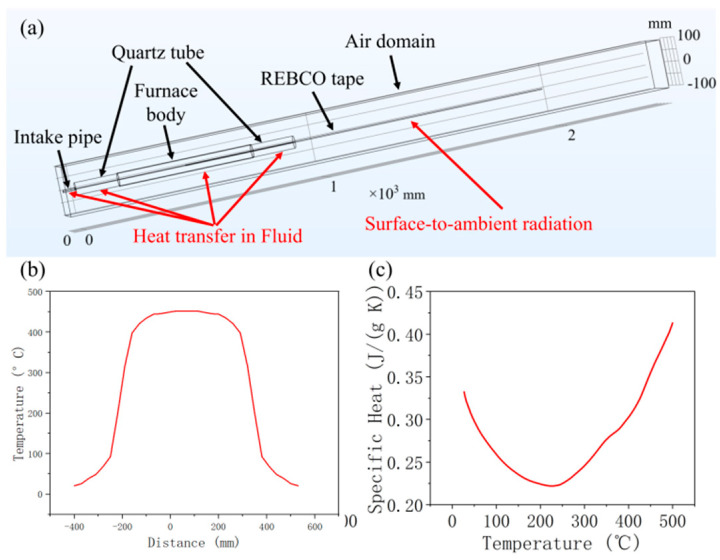
(**a**) Model of tube furnace and REBCO tape; (**b**) temperature profile of inner surface of quartz tube; and (**c**) specific heat profile of REBCO tape.

Figure 4a shows the tube furnace and REBCO tape model established with COMSOL. The model can be found in the Supplementary File. The long REBCO tape was inserted into a quartz tube, whose middle part was surrounded by the furnace body. The furnace body was set to thermal insulation, which prevented the heat from being transmitted to the ambient environment from this part. The intake pipe was set to introduce the flow of gas into the quartz tube. All the components were surrounded by an air domain, with the edge set to a constant temperature of 20 °C. The intake pipe and the quartz tube were set to use heat transfer in both the solid and fluid, with an oxygen flow rate of 60 mL/min, and all the other parts only used heat transfer in the solid. The REBCO tape outside the quartz tube was set to use surface-to-ambient radiation to simulate radiant heat. It is worth mentioning that the current fluid model is quite simple and that fluid–solid coupling is ignored. A different fluid model would definitely bring different heat transformation results. The current simple fluid model only fit the present initial investigation, and a better fluid model should be researched in the future.

Simulating a real furnace was too complex for the heat input part, since heat transfers from the heating wire to the quartz tube and then to the REBCO tape. Since the volume of the REBCO tape was small, we assumed that the temperature of the quartz tube did not change, regardless of whether or not the REBCO was inserted into the furnace. Therefore, we directly used the temperature of the inner face of the quartz tube from the measurements as the heat input, as shown in Figure 4b. It is worth mentioning that, due to the length limitation of the thermocouple, only half of the quartz tube was measured, and the other half used the mirror value. For the material parameters, only the value of REBCO could not be found in the materials database. The measured specific heat of the REBCO tape at different temperatures is shown in Figure 4c. However, the thermal conductivity and surface emissivity values at high temperatures were very hard to measure. More importantly, according to our database, the thermal conductivity of REBCO tape could be very different [21].

**Figure 5:**
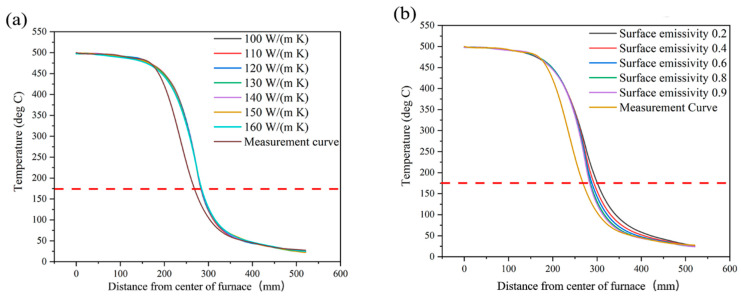
Simulated temperature profiles of REBCO tape with different thermal conductivity values (**a**) and surface emissivity values (**b**). The dashed line is the 175 °C, which is the criterion of the *I*_c_ degradation obtained from Figure 1.

Figure 5 shows the simulation temperature curve of the REBCO tape when using different thermal conductivity values and surface emissivity values. The measured temperature curve was used for comparison. The thermal conductivity of the REBCO tape at room temperature was measured as approximately 160 W/(m K). Since a higher temperature would have decreased the thermal conductivity, values between 100 and 160 W/(m K) were tested, as shown in Figure 5a. Barely any change was found in all of the simulation curves, which indicates that the thermal conductivity of REBCO did not have a noticeable effect. This may have been due to the REBCO cross-sectional area being negligibly small. Regardless of how the thermal conductivity changed, the total heat flux did not exhibit a large difference. The surface emissivity could be varied between 0.2 and 0.9 due to the varying surface radiance. Figure 5b shows that the higher surface emissivity was closer to the measured curve. Although the best combination of thermal conductivity (160 W/(m K)) and surface emissivity (0.9) was used, the simulation curve still showed some differences from the measured curve. The reason for this will be discussed in the next section. Compared with the measured curve, the simulation curve overestimated the temperature at the same distance. In other words, the simulation condition needed a longer distance (16 mm) than the actual measurement condition to maintain the 175 °C threshold. Additionally, in the real tube furnace, the Cu layer removal length was 273 mm, but it was 289 mm in the simulation. Of course, the further optimization of the model is needed for it to more closely match the real condition. However, the longer distance in the simulation results would allow for a greater REBCO tape safety to be achieved, representing a safety margin in the furnace design. We believe that this is acceptable in the current study.

**Figure 6:**
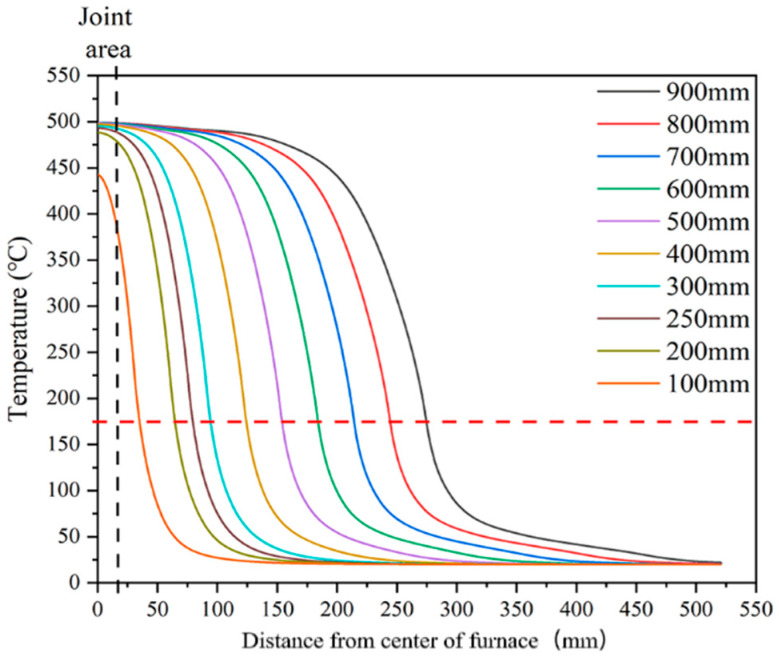
Simulation temperature profiles of REBCO tape with different furnace designs. The dashed line is the 175 °C, which is the criterion of the *I*_c_ degradation obtained from Figure 1.

We then optimized the furnace design based on the current furnace model and two criteria. The first criterion was keeping a spatial range of 20 mm at high temperatures; this was the joint area that needed oxygen compensation. The second one was making the spatial range of temperature over 175 °C as small as possible. The primary protocol involved shortening the length of the furnace, that is, the quartz tube length in our model [20,21,22,23,24,25]. We assumed that the inner quartz tube’s surface temperature curve proportionally decreased with the quartz tube length. Figure 6 shows the simulation temperature curve of the REBCO tape with different furnace designs. According to the two criteria, the 200–300 mm length seemed to be the optimal range, which is far shorter than the current 900 mm. The distance over 175 °C, namely the Cu removal length, was 63–94 mm, only 23–34% of the 273 mm shown in Figure 3. A much shorter Cu removal length can bring two significant advantages: first, a shorter length means a more compact magnet design; second, less Cu removal can lead to better thermal conductivity, which improves the stability of the joint [26]. However, too short of a furnace length also makes it difficult to keep a homogenous temperature distribution at the joint. For example, the temperature range in the joint area (20 mm) was less than 1 °C (499.15–498.42 °C) for the 900 mm furnace. However, for the 200, 250, and 300 mm lengths, the temperature differences were 15.75, 7.45, and 4.33 °C, respectively. The oxygen compensation temperature can be varied in a relatively large area. However, whether a 15 °C temperature difference in the joint area is acceptable needs further investigation.

## 4. Discussion

In the above section, we showed a tube furnace design method for a superconducting joint with a minimal Cu layer removal length. However, three topics need to be further discussed. First, the reason for the difference between the simulation and real measurement values should be explored. Second, the potential application scenarios of the furnace design are discussed based on a literature search. Third, one of the key components of the tube furnace, the flange, is briefly discussed.

As shown in Figure 5, the final simulation result still differed from the actual measurement curve. The most likely reason for this is that we ignored some radiative heat transfer. The primary part should be the tube quartz outside of the furnace in the export direction. The radiative heat transfer in this part was quite complex, especially in the transparent quartz tube. The other possible ignored radiative heat transfer was that along the axis direction of the furnace. We set the furnace part as heat insulation in the radial direction. However, there is still a possibility that radiation heat transferred along the length direction.

In the introduction, we mainly mentioned the application of the furnace in a superconducting joint of REBCO tape. Here, we briefly discuss all the potential application scenarios. Table 2 summarizes the annealing parameters of the different joints from the literature. Similar to the superconducting joint of REBCO, the Bi2223 superconducting joint also needs oxygen annealing [27]. Moreover, the Ag diffusion method is used to fabricate REBCO joints with a lower resistance than that of a conventional solder joint [28,29,30,31,32,33,34,35]. All of these joints need as short of a Cu removal length as possible, which our furnace design could help with. The literature shows that oxygen annealing focuses at 350~500 °C with a time of 1~350 h. We initially thought that our furnace design model could fit this temperature range. However, the fabrication of a REBCO superconducting joint also requires a higher-temperature heat treatment, typically in the range of 800~900 °C, with a short time of one minute to tens of minutes. We do not believe that our furnace design can fit the fast rising and cooling or even the atmospheric change requirements of such high-temperature heat treatments.

**Figure 7:**
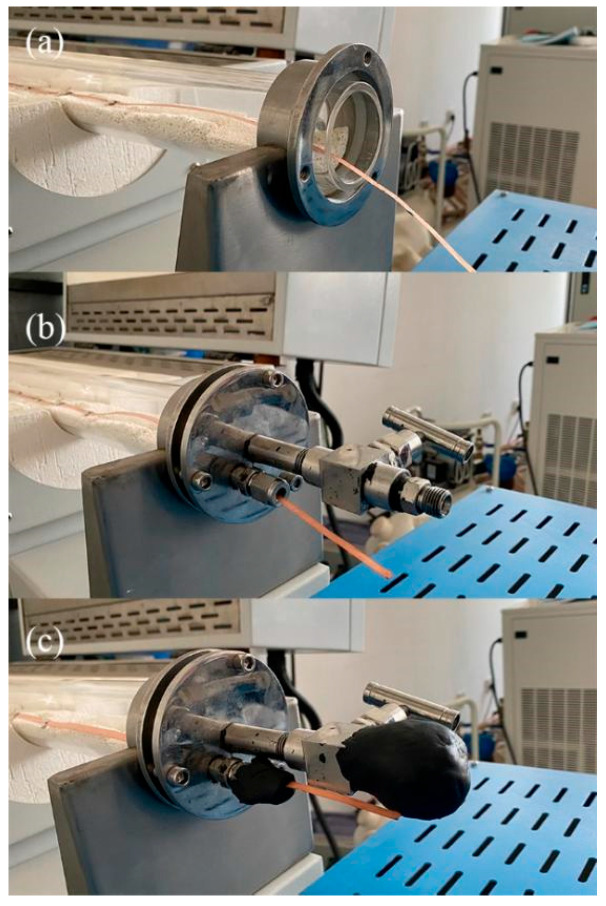
The different kinds of tube furnace ends: (**a**) fully open end; (**b**) flange without seal; and (**c**) flange with seal.

**Figure 8:**
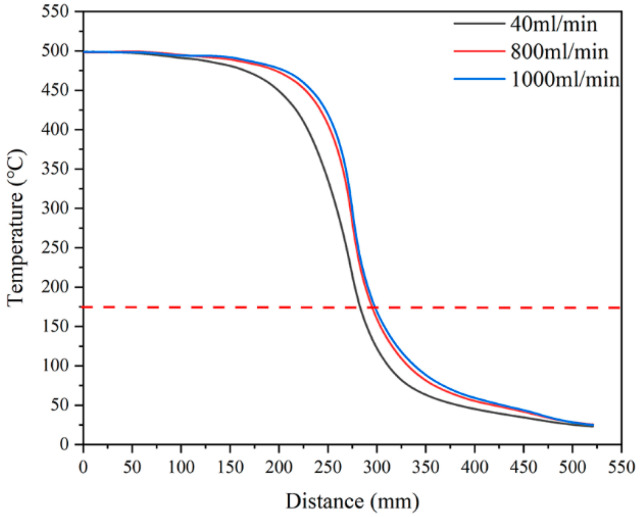
Temperature distribution curves with different flow rates. The dashed line is the 175 °C, which is the criterion of the *I*_c_ degradation obtained from Figure 1.

Besides the length of the tube furnace, the other key component is the flange. On the one hand, oxygen annealing requires a flowing oxygen atmosphere. On the other hand, the superconducting joint is an extension of the large superconducting coil, which means that the flange must have a path for the REBCO tape. Figure 7 shows three different kinds of tube furnace ends, including fully open ends, a flange without a seal, and a flange with a seal. Table 3 shows the relationship between the flow rate and the oxygen fraction when using different flanges. The oxygen content was measured by a high-temperature oxygen concentration detector (RSCORRECT FG100M, Wuhan, China). The detector was put in the middle of the tube furnace where the sample was. It was found that the fully open end had difficulty in keeping an oxygen fraction over 90%, even with an extremely high oxygen flow rate. The flange without a seal could keep a 99% oxygen fraction by using at least 800 mL/min, which is still very high. A high flow rate means a high oxygen consumption. Alongside a high oxygen consumption, a high flow rate also causes a change in the temperature distribution of the REBCO tape. Figure 8 shows the temperature distribution curves with different flow rates. It was found that the length with a temperature over 175 °C became larger with a higher flow rate. This was because the flowing gas can improve the heat exchange in the tube furnace. However, we need to suppress the heat exchange. In contrast, if the flange is sealed, it can easily keep a pure oxygen atmosphere at a 40 mL/min flow rate. However, a well-sealed flange is hard to design and implement. Therefore, the trade-off among ease of use, a high oxygen consumption, and a longer Cu removal length is one of the critical points that needs to be discussed in every furnace design based on different application scenarios.

Finally, we want to discuss the application scenarios of our designed furnace. In this study, we focused on the goal of designing a furnace for the oxygen annealing of superconducting joints. However, as mentioned in the Introduction section, the fabrication of a superconducting joint also needs high-temperature sintering. Hence, there is the possibility that we can design a furnace that allows for joint fabrication and oxygen annealing to be completed in only one furnace by using the current model. The boundary conditions of the furnace design for joint fabrication are the same as that for oxygen annealing; that is, the joint part of the REBCO tape should be kept at a high temperature and the temperature of the other part should be made to decrease below 175 °C as fast as possible. Further work will be conducted in the future.

## 5. Conclusions

In this study, we established a furnace design method for the oxygen annealing of a REBCO superconducting joint. REBCO with a Cu layer was confirmed to have a damage temperature threshold of 175 °C under oxygen. The Cu layer should be removed from all parts of the REBCO tape over this temperature during annealing. The real temperature distribution curve of REBCO tape in a commercial tube furnace was measured. A heat exchange model was also established to simulate the heat change process between the REBCO tape, the furnace, and the ambient environment. According to a comparison of the measurement and simulation results, the length at a temperature over 175 °C in the simulation was slightly longer than in the real measurement, but was acceptable based on our analysis. We optimized the furnace design based on the model according to two criteria: a length of 20 mm of REBCO tape should be kept at high temperatures and the length of tape at temperatures over the temperature threshold should be as short as possible. The optimized furnace length was approximately 200–300 mm, which caused the Cu layer removal length to be only 23–34% of our current 900 mm long furnace. The possible reasons for the discrepancy between the real measurement and the simulation, the potential application scenarios of the tube furnace, and the design of the flange are also discussed.

## Figures and Tables

**Table 1:** The *I*_c_ values of the REBCO tape after sintering.

Sintering Temperature (°C)	*I*_c_ Value After Sintering in Oxygen (A)	*I*_c_ Value After Sintering in Air (A)
Original	127.3	130.1
100	126.6	132.3
150	127.1	124.6
175	123.6	124.9
200	82.5	87.8

**Table 2:** A summary of the annealing parameters of different joints from the literature.

Reference	Temperature (°C)	Time (h)	Atmosphere
REBCO superconducting joint			
[14]	450	2	O_2_
[7]	500	350	O_2_
[15]	500	10	O_2_
[10]	500	6	O_2_
[16]	350–550	15–100	O_2_
[17]	500	350	O_2_
[18]	500	350	O_2_
[22]	770	1	O_2_
REBCO Ag diffusion joint			
[23]	500	50 or 100	O_2_
[24]	400	1	O_2_
[25]	350–400	1	O_2_
[26]	400	3	O_2_
[36]	400	2	O_2_
[37]	500	1	O_2_
[28]	500	1	O_2_
[29]	350–400	1	O_2_
Bi2223 superconducting joint			
[30]	350	10	O_2_

**Table 3:** Relationship between flow rate and oxygen fraction when using different flanges.

Flow Rate (mL/min)	Maximum Oxygen Fraction (%)	Oxygen Fraction After the Reading Is Stable (%)
Fully open end		
400	97	81
600	99	85
800	100	89
1000	100	91
1200	100	91
1400	100	90
Flange without seal		
400	100	89
600	100	93
800	100	99
1000	100	99
Flange with seal		
40	100	100

## Data Availability

The original contributions presented in this study are included in the article/Supplementary Materials. Further inquiries can be directed to the corresponding author.

## Supplementary Materials

The following supporting information can be downloaded at: https://www.mdpi.com/article/10.3390/ma18133053/s1, Supplementary File: Heat exchange model.
